# Hot Forging of DIN 8555 E6-UM-60 Alloy Produced by Directed Energy Deposition: Understanding the Metallurgical Effects

**DOI:** 10.3390/ma19020373

**Published:** 2026-01-16

**Authors:** Carlos Antônio Ferreira, Lirio Schaeffer, Anderson Daleffe, Henrique Cechinel Casagrande, Gilson de March, Joélson Vieira da Silva

**Affiliations:** 1 Department Metallurgical Engineering, Universidade Federal do Rio Grande do Sul (UFRGS), Porto Alegre 90010-150, Brazil; carlos.ferreira@satc.edu.br (C.A.F.); schaefer@ufrgs.br (L.S.); 2 Department Additive Manufacturing, Centro Universitário SATC (UNISATC), Criciúma 88805-380, Brazil; anderson.daleffe@satc.edu.br (A.D.); gilson.march@satc.edu.br (G.d.M.); joelson.silva@satc.edu.br (J.V.d.S.); 3 Department Metallurgical Engineering, Universidade Federal de Santa Catarina (UFSC), Florianópolis 88040-900, Brazil

**Keywords:** hot forging, additive manufacturing, 3D printing, rapid prototyping, microstructure, chromium carbides

## Abstract

This study investigates a hybrid processing route that integrates localized fusion-based additive manufacturing and hot forging for the production of complex-shaped components, with emphasis on metallurgical integrity and mechanical performance. The DIN 8555 E6-UM-60 alloy, traditionally classified as martensitic and applied under severe wear conditions, exhibited atypical metallurgical behavior during hybrid processing, notably the consistent formation of chromium carbides under specific thermomechanical conditions. Metallographic analyses, microhardness measurements, thermographic monitoring, hot tensile tests, and room-temperature tensile tests were performed to establish correlations between microstructure, thermal history, and mechanical response. Specimens produced by additive manufacturing and subsequently hot forged showed a significant reduction in porosity, improved microstructural homogeneity, and partial retention of hardening phases, enabling discussion of recrystallization mechanisms, phase stabilization, and precipitation phenomena in martensitic alloys processed by additive manufacturing. Hot tensile tests revealed limited hot workability of the alloy, while room-temperature tensile tests led to premature fracture, with failure consistently initiating at pre-existing microcracks formed during the forging stage. Although detrimental, these microcracks provide valuable insight into critical processing conditions and ductility limits of the material. Overall, the hybrid route demonstrates strong potential for industrial applications, highlighting the importance of precise thermomechanical cycle control to mitigate defects and enhance structural reliability.

## 1. Introduction

Additive manufacturing (AM) has emerged as an innovative technology for the production of a wide range of components using various materials, offering significant advantages in terms of design flexibility, waste reduction, and optimization of tooling usage [1,2]. In addition, recent studies have explored the feasibility of bimetallic joining via AM, employing AWS A5.36 E110C-G M steel produced by wire arc additive manufacturing (WAAM) and rolled ASTM B221 6060 aluminum, with the joint evaluated through forging processes [2]. Applications involving stainless steels also warrant attention, particularly due to cost reductions when compared with more expensive alloys, such as austenitic AISI 310 steel [3,4]. Nevertheless, studies focusing on post-processing techniques, such as hybrid AM followed by forging, have gained prominence owing to the substantial reduction in raw material costs, while also offering improved sustainability from an industrial perspective [5,6].

Thus, in forging processes, one of the key challenges faced by industry is the design of preforms with geometries properly tailored to the final component, aiming to eliminate or minimize subsequent machining operations and thereby increase process efficiency [7,8]. Preform design is a critical step that plays an essential role in the quality control of forged products, particularly in near-net-shape forming of forgings with complex geometries [9,10]. To ensure complete filling of the die cavities without defects, as well as to maintain material integrity, one or more preforming stages are commonly employed in the forming operations of complex forgings [11,12,13,14].

The application of additive manufacturing to the production of preforms for forging emerges as a promising solution, as it enables the fabrication of components with optimized material distribution and a reduced number of intermediate forging stages. Technologies such as wire arc additive manufacturing (WAAM) allow the production of a wide range of geometries, promoting more efficient use of raw materials and enhanced overall efficiency of the forging process [15,16,17,18].

The integration of additive manufacturing (AM) and hot forging has been extensively investigated as an effective strategy to overcome inherent limitations of components produced solely by AM. While AM enables the fabrication of complex geometries and near-net-shape preforms, parts manufactured by fusion-based processes often exhibit residual porosity, mechanical anisotropy, chemical microsegregation, and non-equilibrium microstructures, resulting from high thermal gradients and rapid solidification rates. Experimental studies have demonstrated that forging applied as a post-processing step can significantly reduce porosity, promote grain refinement through dynamic recrystallization, homogenize the microstructure, and improve the overall mechanical response of AM-produced alloys, as observed in 316L stainless steel and other structural alloys [19,20].

In addition, several hybrid manufacturing routes combining additive manufacturing and mechanical forming have been proposed and evaluated in the literature, involving processes such as laser powder bed fusion, directed energy deposition, and arc-based deposition, followed by forging or rolling. Recent studies show that these hybrid routes enable the attainment of denser microstructures with reduced anisotropy and more isotropic mechanical properties compared with those obtained by AM alone, particularly in titanium alloys and high-strength steels [21]. Comprehensive reviews emphasize that the success of these approaches strongly depends on precise control of the thermomechanical cycle, processing sequence, and the compatibility between the microstructure formed during deposition and the deformation mechanisms activated during forging [22].

This article aims to investigate the mechanical characteristics associated with the chemical composition and the formation of carbide-rich phases of the DIN 8555: E 6-UM-60 alloy [23] through microhardness testing, metallographic analysis, optical emission spectrometry, and hot tensile testing. The impacts of additive manufacturing on the quality of the preforms and on the feasibility of their application at an industrial scale are discussed.

The development of a preform involves design aspects such as modifications to billet geometry, variations in thickness and width, and increased edge radii to reduce forging loads, while ensuring complete filling of the die impression. An appropriate preform design ensures adequate metal flow within the die cavity [7,24,25,26,27,28]. The elimination of flash formation during hot forging not only reduces material loss but also contributes to lower energy consumption, due to reduced preform heating requirements, and shorter processing times associated with flash removal. Furthermore, flashless forging preserves the continuity of microstructural flow, resulting in enhanced mechanical strength of the forged components [29].

The selection of an appropriate hardfacing material requires knowledge of both the tool material and the service environment [30]. In this study, a hardfacing alloy recommended for components subjected to abrasive wear, characterized by high hardness and impact resistance [31,32], was produced by additive manufacturing and subsequently subjected to hot forging to evaluate its feasibility and performance. Visual inspection, metallographic analysis, and tensile testing were performed on the printed material to assess structural integrity and phase distribution after hot forging.

## 2. Materials and Methods

### 2.1. Geometry of the Sample Parts

**Figure 1:**
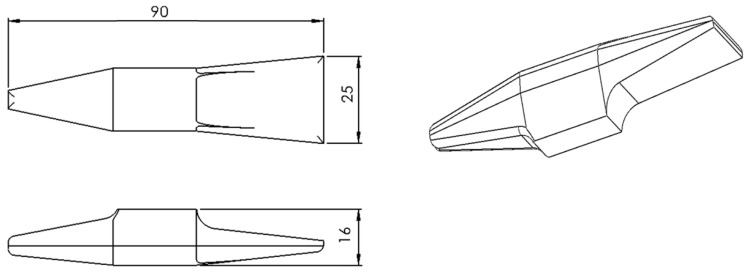
Intended geometry of the sample part after forging.

The geometry was defined with the intention of obtaining a component based on the dimensions of a cross-peen hammer, as shown in Figure 1. The adoption of this model was due to the availability of the forging die.

### 2.2. Printing of the Samples

**Figure 2:**
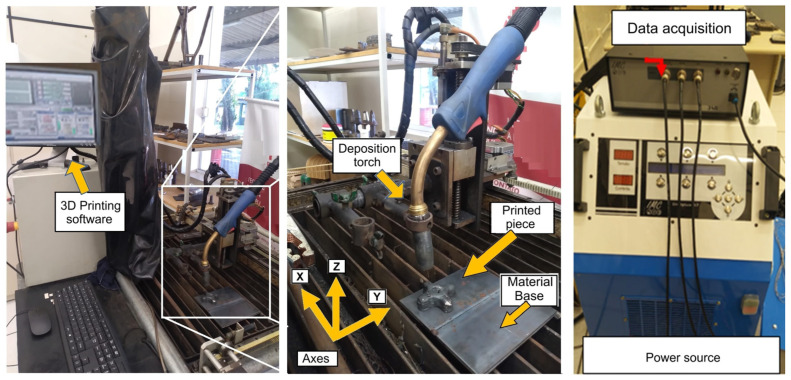
Welding system used.

For the fabrication of the test pieces, samples were prepared using a welding machine adapted by UniSATC. The equipment consists of a shaft coupled to a table with degrees of freedom in the X, Y, and Z axes, operating in an automated manner. To achieve this, a numerical control system was implemented, enabling linear control of the torch height and ensuring greater uniformity in material deposition. The process employed a semi-automatic welding machine, model SMASHWELD 250E (ESAB) (Contagem, Minas Gerais, Brazil), which uses the MIG/MAG (Metal Inert Gas/Metal Active Gas) welding technique. In this study, the MAG welding process was specifically applied. The details of the equipment are shown in Figure 2.

**Figure 3:**
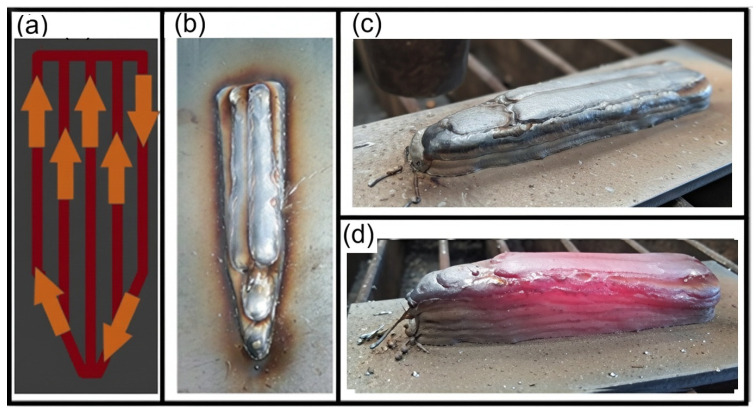
Deposition strategy and toolpath direction (**a**), printed sample (**b**,**c**), sample immediately after printing (**d**).

During the sample printing process, the deposition strategy was evaluated considering factors such as surface finish and material flow, which are influenced by the high thermal energy rate involved. Based on this analysis, the strategy illustrated in Figure 3 was deemed the most appropriate, initiating material deposition at the periphery and concluding with the filling of the central region of the part. Eight deposition layers were required on ASTM A36 steel plates to achieve the target geometry specified in the design.

To monitor the deposition data, the IMC DIGplus A7 welding equipment (Palhoça, Santa Catarina, Brazil) was employed for raw material deposition. The SAP 3SR tool enabled the recording of parameters such as voltage, current, gas flow rate, wire feed speed, among others used throughout the sample fabrication process.

The deposition material used for manufacturing the test specimens consisted of AWS A5.28 UTP AF DUR 600 wire with a diameter of 1.2 mm, and a shielding gas cylinder SG-AC-15s containing a mixture of active gas with 15% carbon dioxide (CO_2_) and 85% argon (Ar). Table 1 presents the data related to the material printing configuration.

A higher gas flow rate for the DUR 600 wire was employed, as recommended by the manufacturer and in accordance with the EN ISO 14175 standard [33].

### 2.3. Hot Forging

The forging process was carried out in a single stage using a press manufactured by FKL (São Leopoldo, Brazil), with a capacity of 150 tons. The tool displacement speed was 12.8 mm per second.

**Figure 4:**
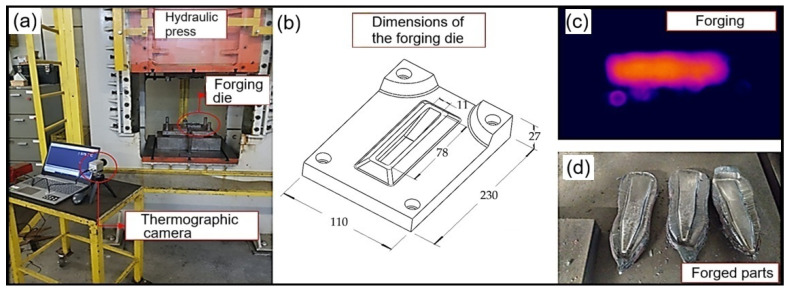
Forging setup (**a**); dimensions of the forging die (**b**); heated preform positioned in the die (**c**); forged samples (**d**).

Forging began with the samples being heated in a muffle-type furnace for a period of 3 h, reaching a temperature of 1150 °C. Figure 4 shows the physical configuration of the equipment used for the forging process and the printed samples.

**Figure 5:**
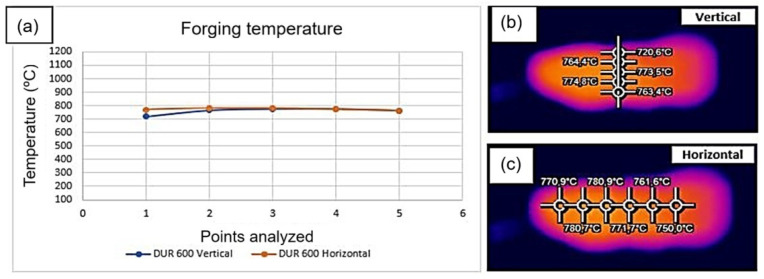
Thermographic analysis of the preform. (**a**) Thermal analysis at the time of forging. (**b**) Temperature values in the longitudinal direction. (**c**) Temperature values in the transverse direction.

The thermal analysis was performed in longitudinal and transversal direction during the forging process. The temperature values for the preforms manufactured by the MA process and the temperature collection points are presented below. The average temperature was 766 °C, as shown in Figure 5.

### 2.4. Thermography of the Printing

To monitor the temperature evolution, an OPTRIS (Berlin, Germany) thermographic camera, model PI 08M, was used, capable of capturing thermal images in the range of 575 to 1900 °C, allowing continuous monitoring and recording of temperature variations during the forging process. Sixteen points were evaluated longitudinally with a spacing of 10 mm, and six points were evaluated transversely, with a distance of 5 mm between them.

### 2.5. Optical Emission Spectrometry

For the optical emission spectrometry (OES) analysis, a BRUKER Q2 ION model spectrometer (Billerica, MA, USA) was used, operating at a power of 400 Watts for 30 s. The sample was initially sectioned using a metallographic cutter, brand Struers, model MESOTOM. Subsequently, it was machined using a Romi U30 milling machine (São Paulo, Brazil) and then ground with a Mello P36 surface grinder (São Paulo, Brazil) in order to reduce surface roughness.

The optical emission spectrometry test was carried out using a BRUKER Q2 ION spectrometer, operated at 400 W for 30 s.

### 2.6. Microhardness Test

The samples were sectioned using a metallographic cutter, brand Struers (Milton, Australia), model MESOTOM. Subsequently, the samples were extracted along the deposition track and underwent grinding with different grit sizes (80, 120, 200, 320, 400, 600, and 1200).

After grinding, the samples were polished using a polisher, brand Fortel (Willenhall, UK), model PFL, with Fortel alumina polishing suspension of 1-micron particle size diluted in water.

**Figure 6:**
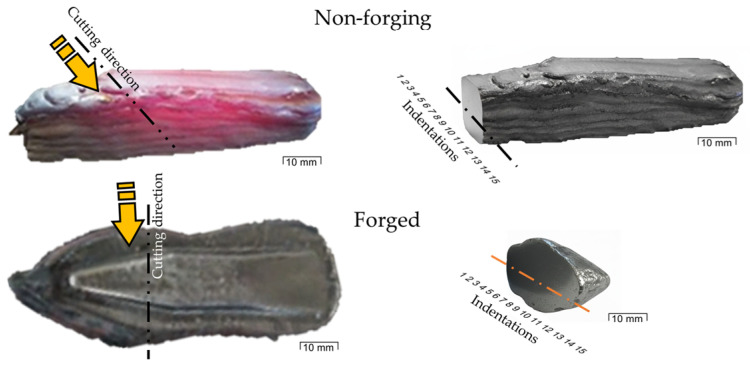
Sample cutting scheme. The lines and arrows indicate the cutting direction.

Hardness homogeneity of the manufactured part was evaluated through a Vickers microhardness test using a SHIMADZU^®^ (Kyoto, Japan) HMV-2TADW microhardness tester. The test was conducted before and after the forging process, under a load of 9.807 N and a dwell time of 10 s. The sample cutting scheme is shown in Figure 6.

### 2.7. Metallographic Analysis

The metallographic analysis was carried out using an Olympus (Tokyo, Japan) SC30–BX 51M microscope, with the chemical composition determined by a Bruker Q2 ION spark spectrometer. Chemical etching of the deposited hardfacing layer was performed using Villella’s reagent (1 g picric acid, 5 mL HCl, and 100 mL ethanol), with an etching time of 180 s.

### 2.8. Hot Tensile Test

**Figure 7:**
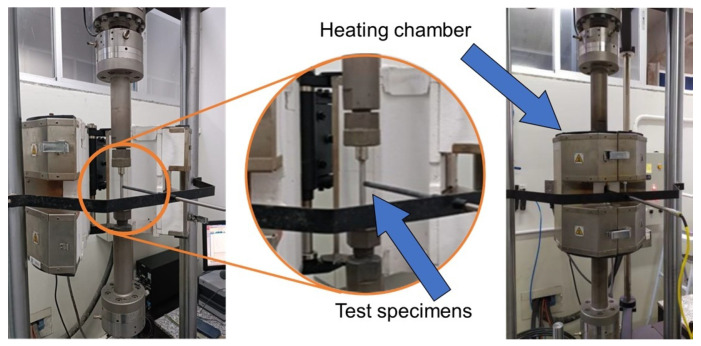
High-temperature tensile testing machine.

The tensile test was performed using the INSTRON^®^ (São José dos Pinhais/Brazil) 8801 equipment, as shown in Figure 7, following the ABNT ISO 6892:2024 [34] standard, at a temperature of 850 °C (±5) with a soaking time of 10 min. Tests were conducted in three directions (0°, 45°, and 90°) at a crosshead speed of 5 mm/min.

**Figure 8:**
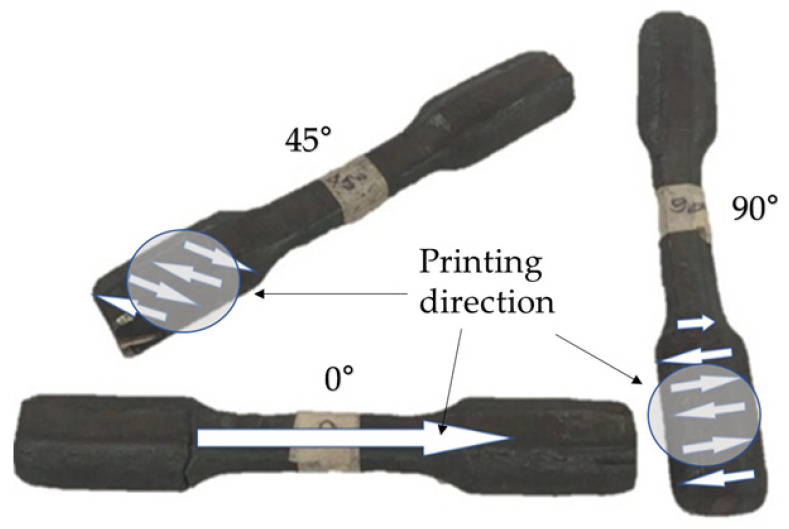
Specimens for tensile testing at elevated temperatures. The detailed views indicate the deposition direction.

The samples after forging, along with an indication of the direction of deposition, can be seen in Figure 8.

**Figure 9:**
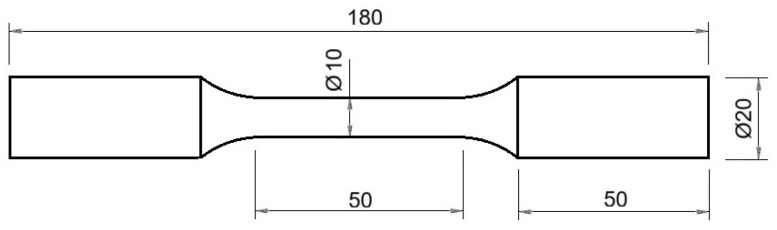
Specimen dimensions after machining.

After machining, the dimensions of the specimen for the high-temperature tensile test were as shown in Figure 9.

The elastic modulus was determined without the use of an extensometer; therefore, the yield strength values obtained using the 0.2% offset method should be regarded as indicative.

### 2.9. Radiographic Testing

Radiographic testing was performed in accordance with the acceptance criteria established in ASME Section VIII, Division 1 [35]. The single-wall single-view (SWSV) technique was employed, using a wire-type image quality indicator (IQI), type 10 FE, in accordance with EN standards [36].

## 3. Results and Discussion

In this study, a preform fabricated from the DIN 8555: E6-UM-60 feedstock, using the localized melting additive manufacturing process, was subjected to hot forging. The aim of this work was to evaluate the mechanical and metallographic properties of a material characterized by high hardness and resistance to surface damage under hot forging conditions.

### 3.1. Thermal Analysis During Additive Manufacturing

**Figure 10:**
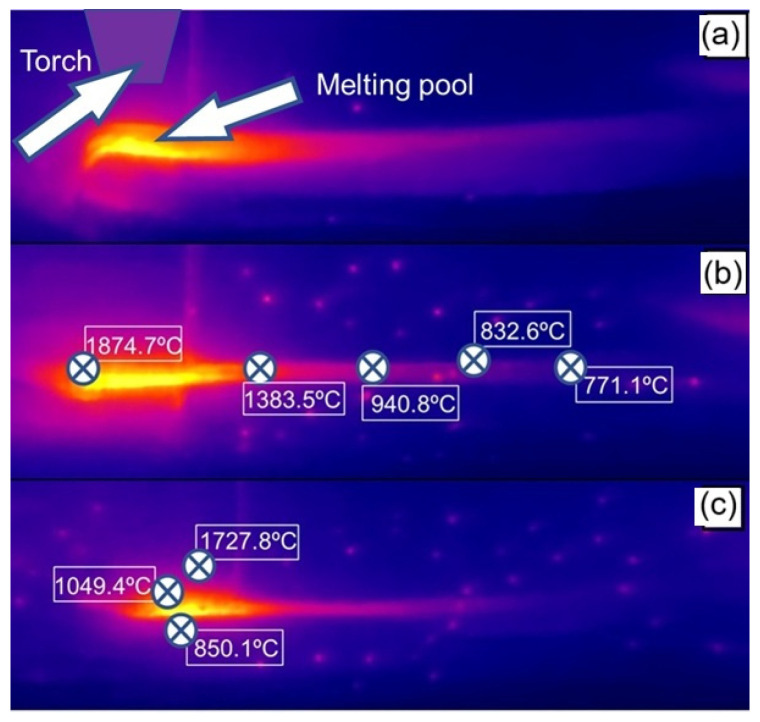
Thermographic analysis of the print. (**a**) Torch and melt pool; (**b**) temperature field along the longitudinal build direction; and (**c**) temperature field along the transverse direction.

Thermal analysis was performed along the longitudinal direction of the part, as indicated in Figure 10b, and transverse analyses were conducted as shown in Figure 10c.

**Figure 11:**
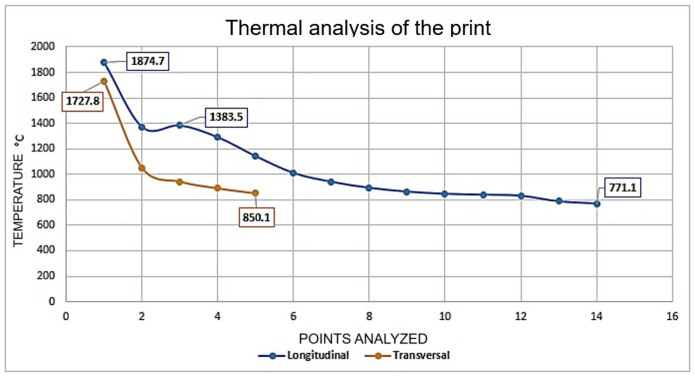
Wire deposition temperatures in longitudinal and transverse directions.

The temperature distribution during the WAAM process reveals the presence of high thermal gradients that are strongly dependent on the position relative to the heat source. As shown in Figure 11, the points analyzed along the longitudinal direction of deposition reached maximum temperatures of approximately 1875 °C in regions closest to the electric arc, followed by a gradual decrease to about 770 °C in more distant regions. This behavior reflects heat dissipation along the deposition path and the associated cooling time.

In contrast, measurements taken transverse to the deposition trajectory exhibited a more pronounced temperature drop, reaching values close to 850 °C. This indicates the existence of significant thermal gradients between the center of the deposited bead and the adjacent zones. Such behavior is characteristic of the WAAM process, in which the high energy density of the arc combined with layer-by-layer deposition promotes complex thermal cycles, involving intense localized heating and relatively rapid cooling outside the molten pool.

Previous studies have reported that these thermal profiles directly influence interlayer heat accumulation, microstructural evolution, and the development of residual stresses. Consequently, they are considered critical factors governing the dimensional stability and mechanical performance of components produced by WAAM [37,38,39,40].

### 3.2. Chemical Analysis

Table 2 presents the nominal chemical composition of the DIN 8555 E6-UM-60 alloy and the experimentally measured values for the WAAM-deposited sample. The measured composition shows good agreement with the nominal specification, with minor variations in the contents of C, Si, Cr, and Mo. Such variations are typical of arc-based welding and deposition processes, in which elemental losses or redistribution may occur due to the high thermal energy involved.

The carbon content of the deposited sample remains close to the nominal value and is sufficient to promote the formation of hardened microstructures, such as martensite and carbides, particularly in alloys containing high levels of carbide-forming elements, such as Cr and Mo. The chromium content plays a key role in enhancing hardenability, wear resistance, and thermal stability of the alloy, while also favoring the formation of carbides, which are widely reported in hardfacing steels and alloys designed for severe service conditions [41,42].

The presence of molybdenum contributes to microstructural refinement and mitigates temper embrittlement, in addition to improving hot wear resistance, an aspect of particular relevance in WAAM processes due to the repetitive thermal cycles involved [43]. Silicon, detected at a slightly higher level than the nominal composition, acts as a deoxidizing element during deposition and contributes to the mechanical strength of the matrix. Manganese assists in stabilizing austenite and reducing weldability-related defects [44].

Overall, the chemical composition obtained is consistent with Fe–Cr–C alloys commonly used in arc-based additive manufacturing processes, indicating potential for the development of hardened microstructures. However, this compositional balance also increases susceptibility to residual stress accumulation and crack formation, especially when combined with high thermal gradients, as reported in previous studies on WAAM of high-hardness alloys [39].

The presence of Mg and Ni may be related to the consumable manufacturing process. Small additions of Mg are commonly used as deoxidizing and inclusion-modifying agents in arc-deposited materials, improving weld pool fluidity and process stability. Nickel may be present as a residual element or as a contributor to weldability.

The non-uniform distribution of chemical elements observed in the material can be attributed to non-equilibrium solidification mechanisms, particularly dendritic microstructure formation. During dendritic growth, alloying elements exhibit different partition coefficients between the solid and liquid phases, leading to solute rejection into the remaining liquid and enrichment of interdendritic regions, a phenomenon known as microsegregation. This behavior is widely reported for metallic alloys solidified under high thermal gradients and non-uniform cooling rates, especially in localized melting processes such as welding and additive manufacturing. As a result, local chemical composition variations may persist after solidification, influencing secondary phase formation, carbide morphology, and the mechanical response of the material [42,45,46].

### 3.3. Metallographic Analysis and Microhardness

**Figure 12:**
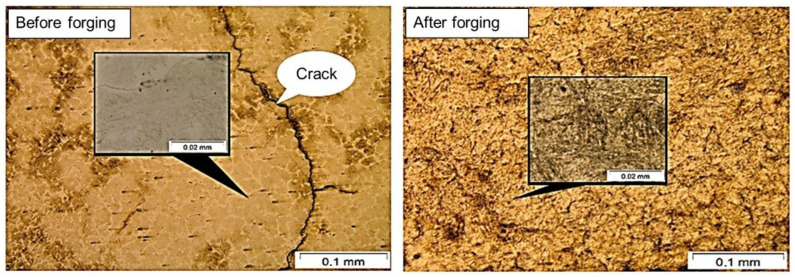
Metallographic analysis of the material before and after forging.

In the metallographic analysis of the material before forging, microcracks were observed, and the structure showed a predominantly ferritic matrix with pearlitic grain boundaries. Regions with pearlite concentrations were also present. The forged sample, on the other hand, exhibited a homogeneous structure with well-defined pearlite concentrations, as shown in Figure 12, at 1000× magnification.

It is clearly observable in the image of the material before forging that crack formed in its matrix, caused by alloying elements such as chromium, which induce internal stresses during deposition [47,48,49]. Nevertheless, in the material after forging, the cracks became less pronounced. A typical martensitic structure was observed, explaining the high hardness. According to Jiang (2022) [50], materials with this chromium content exhibit a typical martensitic structure.

**Figure 13:**
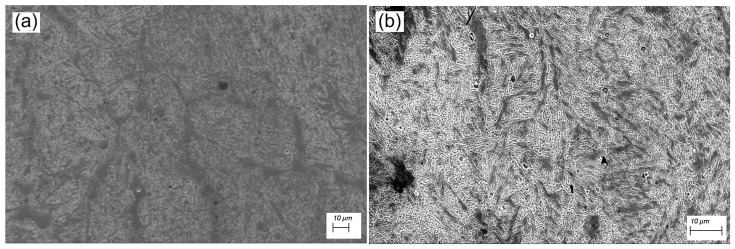
SEM analysis before (**a**) and after forging (**b**).

SEM analyses shown in Figure 13 reveal a relatively coarse and non-refined microstructure, with evidence of undeformed grains and regions exhibiting local elemental segregation. Although no quantitative grain size statistics were obtained, metallographic observations suggest an increase in grain size after forging. This effect is attributed to the deposition process, in which the material enters the austenitic phase and subsequently cools at a moderate rate, resulting in a heterogeneous grain size distribution. According to Chen et al. (2023) [51] and Albannai [52], such moderate thermal cycles, followed by reheating due to subsequent deposition passes, promote annealing and grain growth.

With the forging process at 1150 °C, a more homogeneous and refined microstructure aligned with the deformation flow was obtained, with fewer defects, suggesting a significant improvement in the alloy’s mechanical properties. The observed transition highlights the role of forging in promoting recrystallization, eliminating porosity, fragmenting and redistributing carbides, thereby enhancing hardness and strength, which are critical for mechanically loaded components. A similar condition was reported by Yang (2012) [53] and Costa (2020) [54].

**Figure 14:**
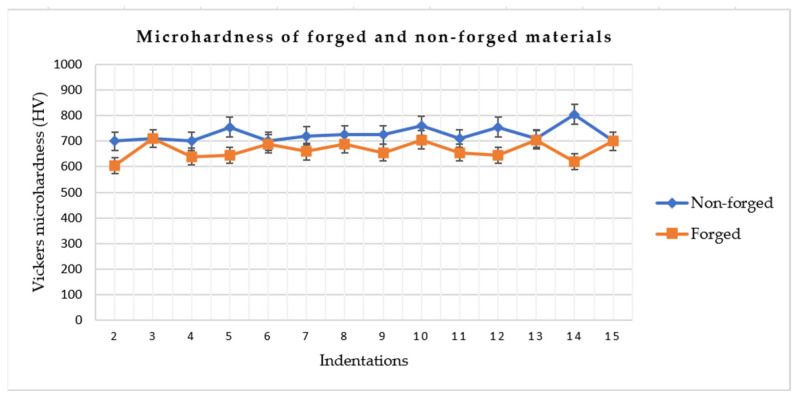
Material microhardness.

The high hardness values were expected due to the presence of alloying elements in the material. In the as-deposited condition, the average hardness was 729 HV with a standard deviation of ±31 HV. After forging, a lower hardness was observed, with an average value of 668 HV and a standard deviation of ±33 HV. This behavior supports the findings of Jiang (2022) [50], who demonstrated that the forging ratio and thermal regime influence carbide distribution and morphology, such that changes in carbide distribution and grain size directly affect hardness. The hardness values obtained for both conditions are presented in Figure 14.

The reduction in hardness may also be attributed to plastic deformation and anisotropy inherited from the thermomechanical history of the material, as well as to phase evolution during the thermomechanical cycle. These effects include partial dissolution of carbides, redistribution of precipitates, and the possible local formation of less strengthening constituents as a result of heating and hot deformation.

In forging processes, particularly when applied to preforms produced by additive manufacturing, plastic flow promotes the alignment of material flow lines, accompanied by grain elongation and reorientation, as well as the redistribution of strengthening phases and crystallographic defects. Although this mechanism contributes to improved structural integrity and directional strength, it may also lead to a local reduction in hardness, especially when partial dynamic recrystallization, carbide coalescence, or relaxation of residual stresses previously introduced during additive deposition occurs.

Studies have shown that the preservation of continuous material flow is directly related to mechanical anisotropy, as the mechanical response becomes dependent on the loading direction relative to grain flow [29]. Similar findings were reported by Jiang et al. (2022) [50], who demonstrated that changes in the thermomechanical regime and forging ratio modify carbide morphology and distribution in martensitic steels, simultaneously affecting hardness and mechanical anisotropy. Furthermore, studies on metallic materials produced by additive manufacturing indicate that microstructural anisotropy resulting from thermal gradients and preferred grain orientation may persist even after thermomechanical post-processing, influencing local hardness measurements and directional mechanical behavior [55]. Therefore, the observed decrease in hardness can be interpreted as the combined effect of microstructural redistribution and flow-induced anisotropy during forging, rather than as a simple degradation of material properties.

### 3.4. EDS Analysis

**Figure 15:**
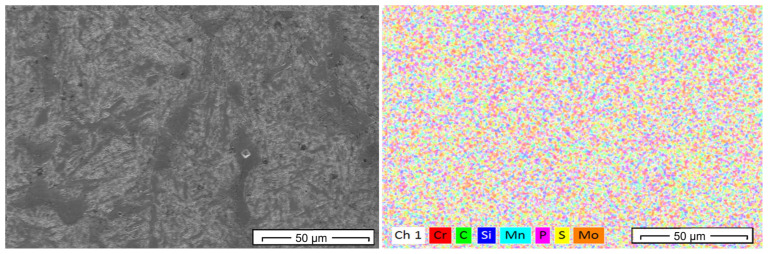
EDS mapping before forging.

Figure 15 shows the EDS mapping prior to forging, revealing an apparently homogeneous distribution of the main elements (Cr, Mo, Mn, Si, and C), possibly resulting from homogenization at 1150 °C for 3 h. However, the EDX spectrum exhibits relatively low intensity peaks, suggesting a low density of fine precipitates or poor dispersion of the alloying elements. The presence of elements such as P and S is still noticeable, which may indicate impurities or segregated inclusions. The Fe peak is not visible in the analysis before forging; however, after forging, the Fe peak becomes more pronounced, which may reflect a higher proportion of the ferritic matrix following structural refinement.

**Figure 16:**
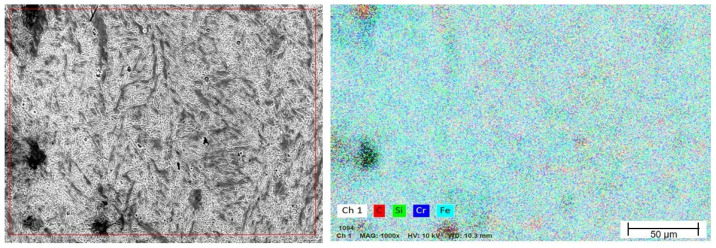
EDS mapping after forging.

The EDS mapping after forging confirms an improved distribution of the alloying elements, particularly Cr and Mo, suggesting partial phase dissolution and redistribution during hot deformation (Figure 16). The presence of Cr-rich carbides is explained by the combination of a significant C content (0.5%) and Cr (5.7%), together with the micro-segregation inherent to the localized melting additive manufacturing process. Heating and deformation at 800 °C promote partial dissolution, fragmentation, and redistribution of carbides; however, due to the thermodynamic stability of Cr-rich carbides, a fraction remains after forging, as confirmed by the EDS maps and SEM images. A similar condition was reported by Martins (2014) [56] and Zhang (2020) [57].

**Figure 17:**
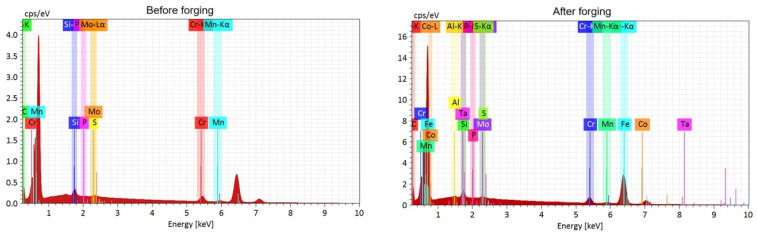
DS spectrometry before and after forging.

**Figure 18:**
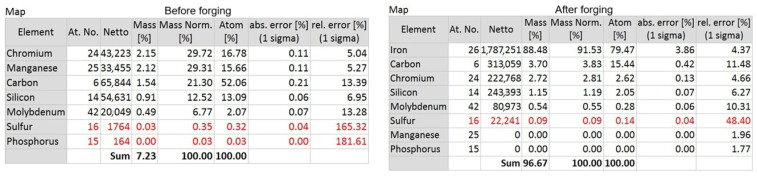
Qualitative microanalysis of chemical elements in the sample.

After forging, the EDX spectrum shows more intense peaks for Fe, Cr, Mn, and Mo, along with the appearance of elements such as Al, Co, and Ta, likely originating from surface contamination, as observed in Figure 17 and Figure 18.

The absence or reduction in the intensity of the Fe peak in the EDS spectra obtained after forging does not indicate the elimination of this element from the alloy. This behavior is associated with the local and semiquantitative nature of the technique, as well as the possible overlap of characteristic peaks with other alloying elements and the microstructural evolution induced by thermomechanical processing. Forging promotes the redistribution and fragmentation of carbide-rich phases, resulting in locally alloy-enriched regions, such as those rich in Cr, which tend to dominate the signal detected by EDS. In addition, the interaction volume of the electron beam may encompass multiple phases after microstructural refinement, thereby reducing the relative intensity of the Fe signal. Therefore, EDS was primarily employed for phase identification and evaluation of elemental distribution, and not for absolute quantification. This behavior is widely reported in the literature on scanning electron microscopy and EDS microanalysis, particularly in multiphase and heterogeneous materials [58].

### 3.5. High-Temperature Tensile Test

The average stress values were determined from the engineering stress–strain curves by averaging the stress values obtained from repeated tensile tests conducted under identical conditions, using the nominal cross-sectional area of the specimens.

**Figure 19:**
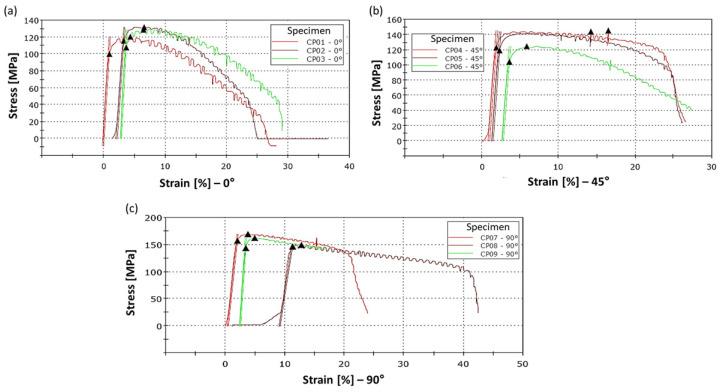
Stress versus Strain Curves for (**a**) Longitudinal (0°), (**b**) Oblique (45°), (**c**) Transverse (90°) directions.

The tensile tests showed an average stress of 127 MPa for the specimens tested in the 0° direction (Figure 19a). In the 45° printing direction, the average stress was 138 MPa (Figure 19b), and in the 90° direction, an average of 160 MPa was obtained, as shown in Table 3 and Figure 19.

Regarding deformation, it can be observed that the printing direction had little influence on the results, with similar outcomes across all three directions. These findings were also reported by Silva et al. (2025) [2]. The tensile curves are shown in Figure 18.

Regarding this test, it can be observed that the stress in the printing direction (0°) exhibited the best mechanical performance, with high strength and ductility. In the 45° printing direction, the performance was intermediate, with lower tensile strength, indicating moderate anisotropy. For the 90° printing direction, the material showed higher strength but was more brittle, with sudden failure occurring in some cases. This behavior, characterized by limited ductility, indicates a reduced ability to accommodate deformation, promoting crack nucleation and propagation under severe thermomechanical conditions, and may be a consequence of the high matrix hardness [59].

Several factors influence the anisotropy of microstructures in metallic components produced by additive manufacturing, which are highly complex. The presence of anisotropy can be linked to various aspects, such as uneven distribution of defects in different directions, crystallographic texture, grain shape, heterogeneous microstructure, and other factors [49]. Analyzing the process conditions, printing parameters significantly affect material anisotropy, including scan rate, layer thickness, scan pattern, and cooling rate [60].

### 3.6. Radiography

**Figure 20:**
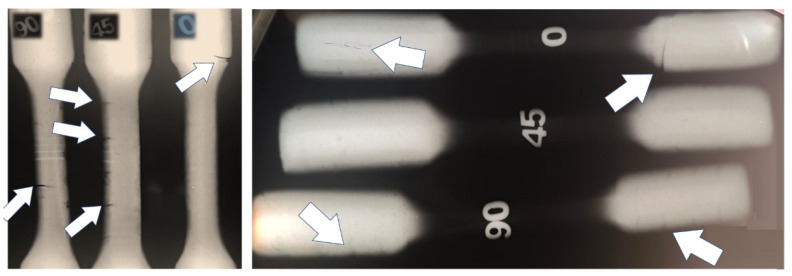
Presence of cracks in the specimens, the most evident cracks are indicated by arrows.

The radiographic evaluation of the forged specimens revealed the presence of linear indications consistent with narrow cracks, predominantly distributed along the specimen length (Figure 20). Radiographic inspection was performed only after forging; therefore, crack evolution could be assessed qualitatively but not quantitatively.

The discontinuities exhibited both longitudinal and transverse orientations, suggesting the combined action of thermal stresses and localized deformation during hot forging. According to Grega et al. (2025) [61], microcracks formed during hot deformation are typical of alloys hardened by phase transformation or precipitation, particularly when subjected to high thermal gradients.

In the case of the DIN 8555 E6-UM-60 alloy produced by localized fusion, the combination of a high martensite fraction, elevated residual stresses inherent to additive manufacturing, and the presence of chromium carbides identified in this study contributes to reduced hot ductility. Similar phenomena have been reported in martensitic steels fabricated by AM, in which the interaction between thermal gradients, layer-induced work hardening, and elemental segregation promotes a behavior prone to hot cracking [62,63]. The concentration of cracks in regions subjected to higher deformation further indicates that the thermomechanical integrity of the deposited microstructure plays a decisive role in the material response during forging.

Regarding the origin of the cracks, microstructural observations of the as-deposited condition revealed pre-existing microcracks, which are commonly associated with solidification phenomena and high residual stresses inherent to arc-based additive manufacturing of martensitic alloys [64]. During the subsequent hot forging stage, these pre-existing defects likely acted as preferential sites for crack propagation under thermomechanical loading [61]. In addition, severe thermal gradients and the limited hot ductility of the alloy may have promoted the nucleation of new cracks during forging, particularly in regions subjected to higher deformation. Further studies on additively manufactured steels indicate that build orientation and heat treatment influence the growth of cracks initiated from microstructural defects [65].

Despite the observed crack formation, hot forging resulted in a significant reduction in porosity and increased microstructural homogeneity throughout the specimens, in agreement with trends reported by Pruncu et al. (2020) [66] for AM components densified by hot deformation. Therefore, the detected cracks should not be interpreted solely as defects but rather as direct indicators of the alloy’s formability limits under the applied thermomechanical parameters. They provide valuable guidance for refining the hybrid processing route, particularly with respect to forging temperature control, component preheating, and reduction of deformation rates—factors identified by Bidare et al. (2021) [67] as critical for minimizing crack formation in AM-deposited alloys.

In summary, the results demonstrate that the hybrid AM–forging route is effective in consolidating and densifying the microstructure of the DIN 8555 E6-UM-60 alloy, although the occurrence of hot cracking highlights the need for further calibration of the thermomechanical cycle. The correlation between the observed cracks, the identified microstructural mechanisms, and the evidence reported in the literature enhances the understanding of the operational limits of this alloy and contributes to the development of more robust processing strategies for applications under severe wear conditions.

## 4. Concluding Remarks

This study demonstrated that the hybrid processing route combining localized fusion additive manufacturing and hot forging represents a promising approach for processing the DIN 8555 E6-UM-60 alloy, particularly with regard to porosity reduction and microstructural homogenization. The analyses showed that forging promoted effective consolidation of the deposited regions, reinforcing the potential of AM–forging integration to enable the production of complex-shaped components with improved metallurgical integrity.

The detection of cracks in the forged specimens, although undesirable from a mechanical standpoint, provided critical insights into the thermomechanical limits of the alloy and enabled the identification of conditions that promote hot cracking. These results are consistent with recent literature, which highlights the interaction between residual stresses, thermal gradients, and hardened martensitic microstructures as key factors for crack nucleation in hybrid processes and post-processing techniques involving additive manufacturing [51,52,56]. Therefore, the observed cracks should be interpreted more as indicators of the intrinsic material behavior under a given processing route than as isolated failures.

The findings also indicate that adjustments to forging parameters, including working temperature, deformation regime, and preheating strategies, are required to enhance the alloy’s formability and mitigate crack formation. A deeper understanding of chromium carbide precipitation mechanisms and the stability of strengthening phases represents an additional important step toward optimizing the thermomechanical response of the DIN 8555 E6-UM-60 alloy in post-processing routes. Such understanding enables the definition of targeted interventions to eliminate defects and increase the alloy’s reliability.

Taken together, the results provide a solid scientific basis for improving AM–forging routes applied to high-performance martensitic alloys. Future studies should focus on thermomechanical process modeling, the evaluation of different geometries and cooling rates, and the implementation of thermal control strategies capable of preventing the nucleation of the identified cracks. These advances may significantly expand the industrial applicability of hybrid and post-processed components for severe wear conditions.

Overall, the results indicate that the application of hot forging as a post-processing technique for additively manufactured preforms led to relevant improvements in microstructural integrity, including reduced porosity and increased microstructural homogeneity, albeit accompanied by limitations associated with the ductility of the investigated alloy. The decrease in microhardness values after forging was attributed to microstructural evolution and residual stress relief, while the observed anisotropic response was related to preferential microstructural orientation and the directional distribution of defects. Hot tensile tests revealed a narrow formability window, consistent with crack initiation and propagation during thermomechanical processing. These findings reinforce the potential of the proposed route for industrial applications, while also highlighting the need for strict control of thermal and mechanical parameters to enhance the structural reliability of the material.

The present study represents an initial investigation into the application of hot forging as a processing technique for additively manufactured materials. Future work is recommended to further elucidate the observed phenomena, including comparative experiments with different heating rates and holding times, as well as the acquisition of quantitative grain size statistics. In addition, the authors acknowledge that performing radiographic inspection prior to forging, combined with quantitative crack density analysis, would enable a more detailed assessment of defect evolution throughout processing. These aspects are considered natural and relevant extensions of the present work and will be addressed in future investigations.

## Figures and Tables

**Table 1:** Additive Manufacturing Data.

Variables	Parameters
Torch travel speed (mm/min)	300
Number of cords	8
Average deposition height (mm)	3
Argon gas (%)	85
CO_2_ gas (%)	15
Gas flow rate (L/min)	20
Wire feed speed (m/min)	4
Voltage (V)	22
Current (A)	160

**Table 2:** Chemical Composition of the Studied Alloy.

Material	C	Mg	Si	Cr	Mo	Mn	Ni
DIN 8555 E6-UM-60	0.60		0.60	6.80	0.50	0.70	
Sample	0.562	1.084	0.966	5.714	0.597		0.047

**Table 3:** High-Temperature Tensile Test Result.

Printing Direction	Average Stress (MPa)	Elongation (%)	Yield Strength (MPa)
Longitudinal—0°	127	28	161
Oblique—45°	138	25	115
Transverse—90°	160	26	149

## Data Availability

The original contributions presented in this study are included in the article. Further inquiries can be directed to the corresponding author.
